# Elephantiasis and Directed Occupational Rehabilitation

**DOI:** 10.1155/2019/6486158

**Published:** 2019-02-11

**Authors:** Jose Maria Pereira de Godoy, Henrique Jose Pereira de Godoy, Ana Carolina Pereira de Godoy, Maria de Fátima Guerreiro Godoy

**Affiliations:** ^1^Cardiology and Cardiovascular Surgery Department of the Medicine School in São José do Rio Preto (FAMERP), CNPq (National Council for Research and Development), Brazil; ^2^Medicine School of Universidade Federal do Mato Grosso-Cuiabá-UFMT and Research Group in the Clínica Godoy, Sao Jose do Rio Preto, Brazil; ^3^Pediatrics Unit Intensive Therapy of Santa Casa de São Paulo, Brazil and Research Group of Clínica Godoy, São Jose do Rio Preto, Brazil; ^4^Medicine School in São José do Rio Preto (FAMERP) and Research Group in the Clínica Godoy, Sao Jose do Rio Preto, Brazil

## Abstract

The aim of the present study was to demonstrate the cure of elephantiasis over a ten-year follow-up period and novel discoveries with directed occupational rehabilitation. A 66-year-old female patient with a history of bilateral lower limb lymphedema reported the aggravation of the condition over the years, reaching stage III (elephantiasis). The physical examination confirmed elephantiasis. The circumference of the left lower limb was 106 cm. Her body weight was 106 kilograms, height was 160 cm, and the body mass index (BMI) was 41.6 kg/m^2^. The patient was submitted to intensive treatment for three weeks, which led to a 21-kg reduction in weight and 66 cm reduction in leg circumference. Ten years after treatment, the patient has maintained the results with the compression stockings. Elephantiasis can be cured, although lymphedema cannot. The cure of elephantiasis depends on maintaining the treatment of lymphedema after normalization or near normalization. Directed occupational therapy stimulates the search for new activities and a life closer to normality.

## 1. Introduction

Lymphedema is specific type of edema in which a failure of the lymphatic system leads to the accumulation of macromolecules in the interstitial space and the retention of fluid [1]. Lymphedema can be congenital or acquired. In congenital cases, the individual is born with an altered lymphatic system that may or may not lead to lymphedema. In secondary cases, there is no problem with the lymphatic system at birth, but events occurring throughout life can cause damage to the lymphatic system, leading to failure and the formation of edema [1, 2].

This condition is divided into three clinical stages. In stage I, the patient awakens without edema, but swelling develops throughout the day. In stage II, the patient awakens with edema, which worsens throughout the day. Stage III (elephantiasis) is a more advanced stage, in which large deformities occur [1–3].

In recent years, a novel concept for the treatment of lymphedema has been developed by Godoy & Godoy aimed at the normalization or near normalization of edema in all clinical stages, including elephantiasis [3]. The researchers have developed novel manual and mechanical lymphatic drainage methods that enable the significant mobilization of macromolecules, thereby affecting the physiopathology of lymphedema [4, 5]. Another novel concept was compression stockings and braces made of grosgrain fabric, which allow a reduction in volume and the maintenance of the results [6]. Intensive, multidisciplinary treatments (eight hours per day) are other accomplishments that enable an approximately 50% reduction in volume in the first five days of treatments [3]. The multidisciplinary approach is based on directed occupational rehabilitation and the development of new skills.

The aim of the present study was to demonstrate the cure of elephantiasis over a ten-year follow-up period and novel discoveries with directed occupational rehabilitation.

## 2. Case Report

**Figure 1:**
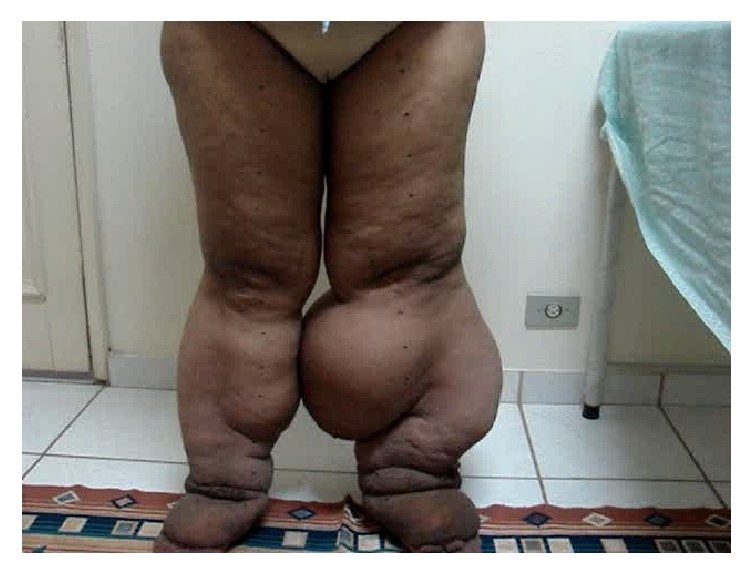
Initial condition before of treatment.

A 66-year-old female patient with a history of bilateral lower limb lymphedema reported the aggravation of the condition over the years, reaching stage III (elephantiasis). The patient was sent to the Godoy Clinic and reported having undergone several treatments throughout her life as well as several episodes of erysipelas. She did not marry due to the lymphedema and complained of the frequent occurrence of strangers staring at her leg, which upset her. The physical examination confirmed elephantiasis. The circumference of the left lower limb was 106 cm. Her body weight was 106 kilograms, height was 160 cm, and the body mass index (BMI) was 41.6 kg/m^2^ (Figure 1).

**Figure 2:**
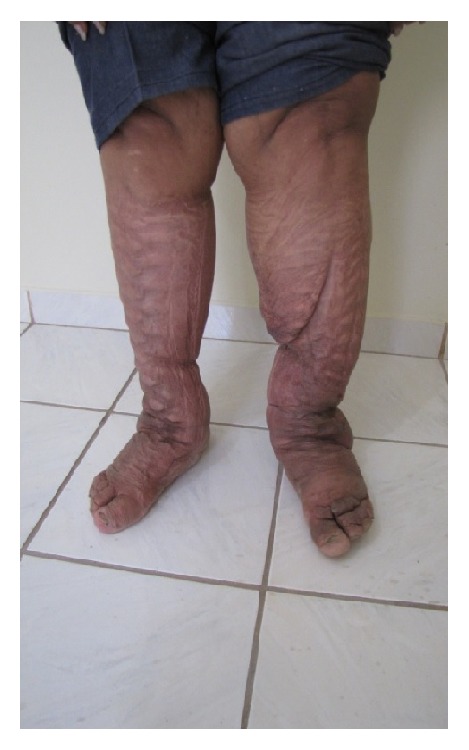
After 3 weeks of treatment.

The patient was submitted to intensive treatment for three weeks, which led to a 21 kg reduction in weight and 66 cm reduction in leg circumference (Figure 2).

Intensive treatment with the Godoy Method consisted of eight hours per day of mechanical lymphatic drainage, 15 minutes of simultaneous cervical lymphatic therapy, and hand-crafted compression stockings made from grosgrain fabric. Mechanical lymphatic therapy consisted of an electromechanical device that performs plantar flexion and extension. After three weeks of intensive therapy, the patient continued treatment at home using the compression stockings. At the follow-up evaluation, the patient was submitted to electrical bioimpedance analysis as well as circumference measurements and volumetry. The bioimpedance analysis revealed a pattern of normality, with the reduction in lymphedema. Ten years after treatment, the patient has maintained the results with the compression stockings. In occupational therapy throughout this period, the patient has been encouraged to perform activities that she has always wanted to do to improve her wellbeing. She took up belly dancing, followed by tap dancing. She reports that these activities changed her life and she is very happy for being able to realize her dream of dancing, which is an activity that she began at the age of 76 years. The study was approving Ethical Committee of Medicine School of Sao Jose do Rio Preto# 2.929.115.

## 3. Discussion

The present study demonstrates that elephantiasis can be cured, as the patient has been without this condition for ten years. She continues to have lymphedema, which ranges from subclinical to stages I and II, varying at different times in her life. She was orientated to improve her quality of life by performing the activities that she had always wanted to do. A certain flexibility in treatment is possible and she is able to forego the use of the stockings on some occasions, such as going to the beach and spending a week without the stockings with little edema. This flexibility enables a better quality of life. After these periods, she returns to correct treatment with the use of the compression stockings.

Another important detail was the identification of whether the patient dreamed of performing any particular activity. In this case, the choice was dance. The dance teacher was instructed by a therapist specialized in directed occupational rehabilitation for patients with lymphedema and they worked together with this purpose in mind [7]. The realization of the lifelong dream of a 76-year-old woman who had had elephantiasis was a source of joy and accomplishment. This aspect shows the extent to which occupational therapy can be useful in the rehabilitation of patients with lymphedema able to change the lives of these people and reestablish the pleasure of living, as reported by the patient in the present case. This opens an opportunity in the field of occupational therapy. There are 16 million people in the world in stage III lymphedema (elephantiasis) and millions others in the earlier stages that could benefit from this approach.

## 4. Conclusion

Elephantiasis can be cured, although lymphedema cannot. The cure of elephantiasis depends on maintaining the treatment of lymphedema after normalization or near normalization. Directed occupational therapy stimulates the search for new activities and a life closer to normality.
